# High prevalence of very-low *Plasmodium falciparum* and *Plasmodium vivax* parasitaemia carriers in the Peruvian Amazon: insights into local and occupational mobility-related transmission

**DOI:** 10.1186/s12936-017-2063-x

**Published:** 2017-10-16

**Authors:** Gabriel Carrasco-Escobar, Julio Miranda-Alban, Carlos Fernandez-Miñope, Kimberly C. Brouwer, Katherine Torres, Maritza Calderon, Dionicia Gamboa, Alejandro Llanos-Cuentas, Joseph M. Vinetz

**Affiliations:** 1 Laboratorio ICEMR-Amazonia, Laboratorios de Investigación y Desarrollo, Facultad de Ciencias y Filosofia, Universidad Peruana Cayetano Heredia, Lima, Peru; 2 Facultad de Salud Pública, Universidad Peruana Cayetano Heredia, Lima, Peru; 3 Division of Epidemiology, Department of Family Medicine & Public Health, University of California, San Diego, La Jolla, CA USA; 4 Departamento de Ciencias Celulares y Moleculares, Facultad de Ciencias y Filosofía, Universidad Peruana Cayetano Heredia, Lima, Peru; 5 Instituto de Medicinal Tropical “Alexander von Humboldt”, Universidad Peruana Cayetano Heredia, Lima, Peru; 6 Division of Infectious Diseases, Department of Medicine, University of California, San Diego, 9500 Gilman Drive MC0760, Biomedical Research Facility-2, Room 4A16, La Jolla, CA USA

**Keywords:** Malaria, Sub-microscopic, *Plasmodium vivax*, *Plasmodium falciparum*, Molecular epidemiology, Serology, MSP10, Sensitivity, Specificity, Migration, Human mobility

## Abstract

**Background:**

The incidence of malaria due both to *Plasmodium falciparum* and *Plasmodium vivax* in the Peruvian Amazon has risen in the past 5 years. This study tested the hypothesis that the maintenance and emergence of malaria in hypoendemic regions such as Amazonia is determined by submicroscopic and asymptomatic *Plasmodium* parasitaemia carriers. The present study aimed to precisely quantify the rate of very-low parasitaemia carriers in two sites of the Peruvian Amazon in relation to transmission patterns of *P. vivax* and *P. falciparum* in this area.

**Methods:**

This study was carried out within the Amazonian-ICEMR longitudinal cohort. Blood samples were collected for light microscopy diagnosis and packed red blood cell (PRBC) samples were analysed by qPCR. Plasma samples were tested for total IgG reactivity against recombinant PvMSP-10 and PfMSP-10 antigens by ELISA. Occupation and age 10 years and greater were considered surrogates of occupation-related mobility. Risk factors for *P. falciparum* and *P. vivax* infections detected by PRBC-qPCR were assessed by multilevel logistic regression models.

**Results:**

Among 450 subjects, the prevalence of *P. vivax* by PRBC-PCR (25.1%) was sixfold higher than that determined by microscopy (3.6%). The prevalence of *P. falciparum* infection was 4.9% by PRBC-PCR and 0.2% by microscopy. More than 40% of infections had parasitaemia under 5 parasites/μL. Multivariate analysis for infections detected by PRBC-PCR showed that participants with recent settlement in the study area (AOR 2.1; 95% CI 1.03:4.2), age ≥ 30 years (AOR 3.3; 95% CI 1.6:6.9) and seropositivity to *P. vivax* (AOR 1.8; 95% CI 1.0:3.2) had significantly higher likelihood of *P. vivax* infection, while the odds of *P. falciparum* infection was higher for participants between 10 and 29 years (AOR 10.7; 95% CI 1.3:91.1) and with a previous *P. falciparum* infection (AOR 10.4; 95% CI 1.5:71.1).

**Conclusions:**

This study confirms the contrasting transmission patterns of *P. vivax* and *P. falciparum* in the Peruvian Amazon, with stable local transmission for *P. vivax* and the source of *P. falciparum* to the study villages dominated by very low parasitaemia carriers, age 10 years and older, who had travelled away from home for work and brought *P. falciparum* infection with them.

## Background

Over the past decade international funding, ongoing political commitment, and improved diagnostic, prevention and treatment strategies have facilitated worldwide reduction of malaria incidence and mortality [1]. Unfortunately in contrast to this trend, the Loreto Region of Peru (which accounts for 97% of the Peruvian countrywide malaria burden) experienced a fivefold increase in malaria cases, as reported through passive surveillance to the Peruvian Ministry of Health (MoH) between 2011 and 2015, reaching a peak of 54,823 *Plasmodium vivax* cases in 2014 and 12,646 *Plasmodium falciparum* cases in 2015 [2, 3].

While the Peruvian Amazon basin has traditionally been classified as a low transmission setting [3–9], foci of high malaria transmission have been identified in some villages across the Loreto Region [10, 11], where the modulation of parasite density by acquired immunity in the host must occur due to repeated infections as is common in high transmission settings [12] or due to clonal infections for long periods within these areas [13–15].

In contrast to patients with acute malaria disease, who generally seek treatment at MoH facilities (thus treatment immediately interrupts transmission, particularly with *P. vivax*), individuals with premunition or “clinical immunity” (i.e. lack of typical symptoms of malaria such as fever, chills, sweats and headache in the presence of low parasite density) do not experience overt systemic inflammation or acute malarial disease. Because such individuals do not present for medical attention and anti-malarial treatment, they remain as potential parasite reservoirs that maintain local transmission with micro-geographic or regional movement of parasites [9, 16, 17], undermining regional control and elimination efforts.

In this scenario, where malaria transmission is maintained by a high prevalence of submicroscopic infections, the development and application of tools to identify these reservoirs is an important priority. This is especially important given that standard malaria control systems in the Americas and elsewhere, including that used by the Peruvian MoH, rely on passive case detection (PCD) of symptomatic infections confirmed only by light microscopy at health facilities [18, 19]. Several studies have suggested that multiple metrics are needed to reflect regional and national trends and current malaria transmission [11, 20–24]. Implementation of these must be guided by the precision, accuracy and cost of each metric [22].

The present study tested the hypothesis that very low parasitaemia of both *P. vivax* and *P. falciparum* is common in contrasting epidemiological contexts of the Peruvian Amazon. Further asymptomatic parasite carriage and human occupation-related mobility across areas with different levels of endemicity leads to local reintroduction of *Plasmodium* infections was assessed. Detailed data to support these hypotheses would be of high generalizable importance in explaining how hypoendemic malaria is maintained on a regional basis. Further such data would support the notion that occupationally mobile, asymptomatically parasitaemic individuals contribute to local movement and regional reintroductions of malaria parasites, making elimination challenging in the absence of targeting anti-malarial interventions at such populations. To address these questions, this study used whole blood samples to estimate an accurate burden of *P. vivax* and *P. falciparum* infections in two villages of the Peruvian Amazon through a parasitological and serological survey.

## Methods

### Study area and population

**Fig. 1:**
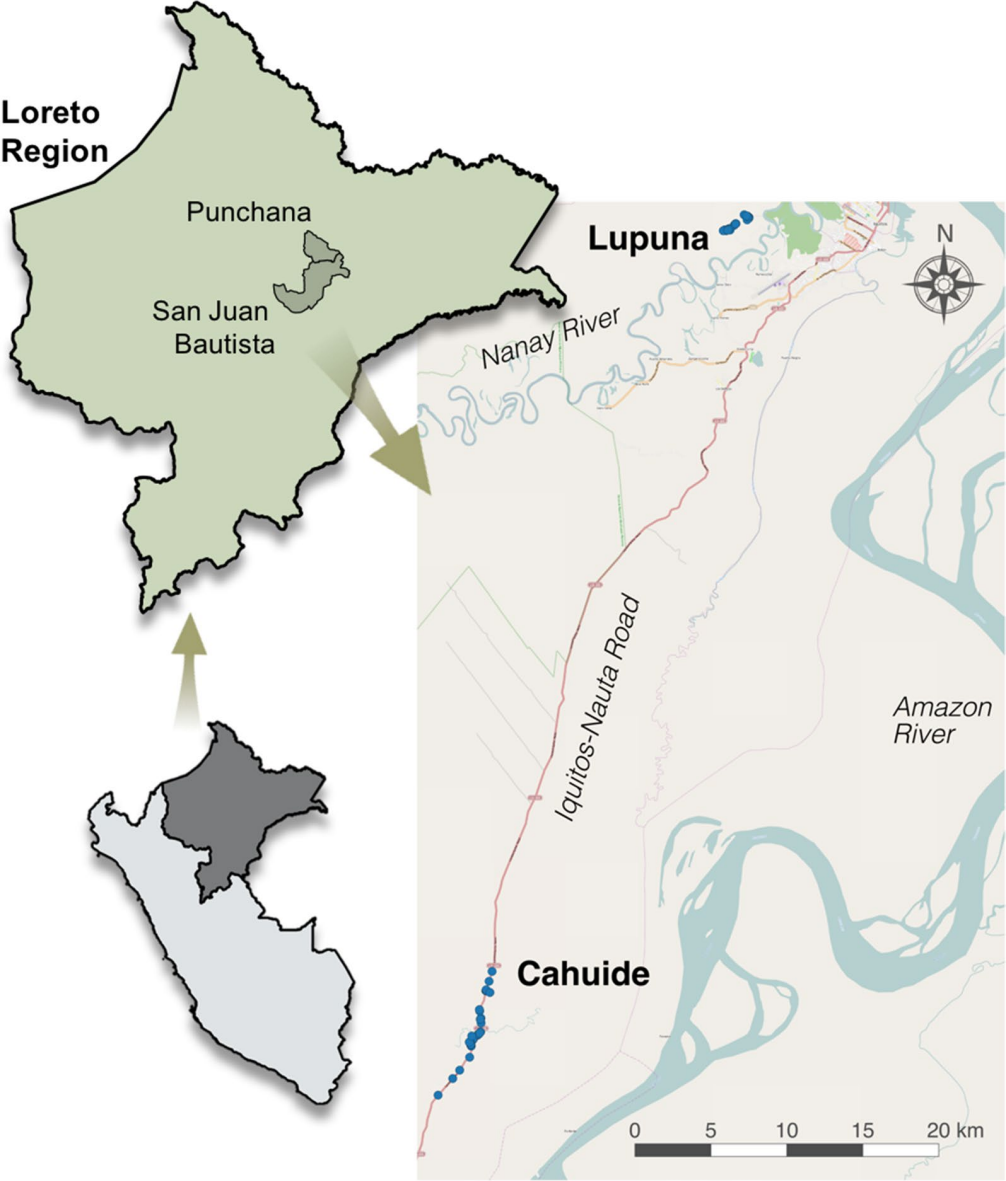
Study sites in Loreto Region. Sampling was carried out in two epidemiologically contrasting sites of the Peruvian Amazon, Cahuide (south of Iquitos city) and Lupuna (north of Iquitos city)

This study was conducted in two sites of Loreto, near the capital city of Iquitos in the Peruvian Amazon: Cahuide and Lupuna (Fig. 1). Cahuide (04°13.785′ S, 73°276′ W) is located 60 km from Iquitos city on the Iquitos-Nauta road in the district of San Juan Bautista (south of Iquitos city). This site is divided into three villages: 12 de April, Cahuide and La Habana, distributed along the Iquitos-Nauta road. Lupuna (03° 44.591′ S, 73° 19.615′ W) is a network of three villages: San José de Lupuna, San Pedro, and Santa Rita, located on the banks of the Nanay River in the district of Punchana (north of Iquitos city); access to this site is only by river. The population of both sites consists mainly of mestizos (individuals that cannot be clearly identified as belonging to a specific ethnic minority but generally being genetic admixture of Hispanic ancestry and indigenous peoples). Living standards are generally based on a subsistence economy including agriculture, fishing and occasional hunting [25, 26].

Malaria transmission in Loreto near to Iquitos has been generally thought to be seasonal. In Lupuna, malaria peaks from November to May, the rainier season [27, 28]. In Cahuide, malaria generally has the same pattern, but, after an outbreak in 2011, has decreased overall [29]. *Anopheles darlingi* is the principal mosquito vector in both villages with diverse breeding sites, including slow moving parts of larger rivers, smaller streams, pools, and swamps [26].

### Study design

**Fig. 2:**
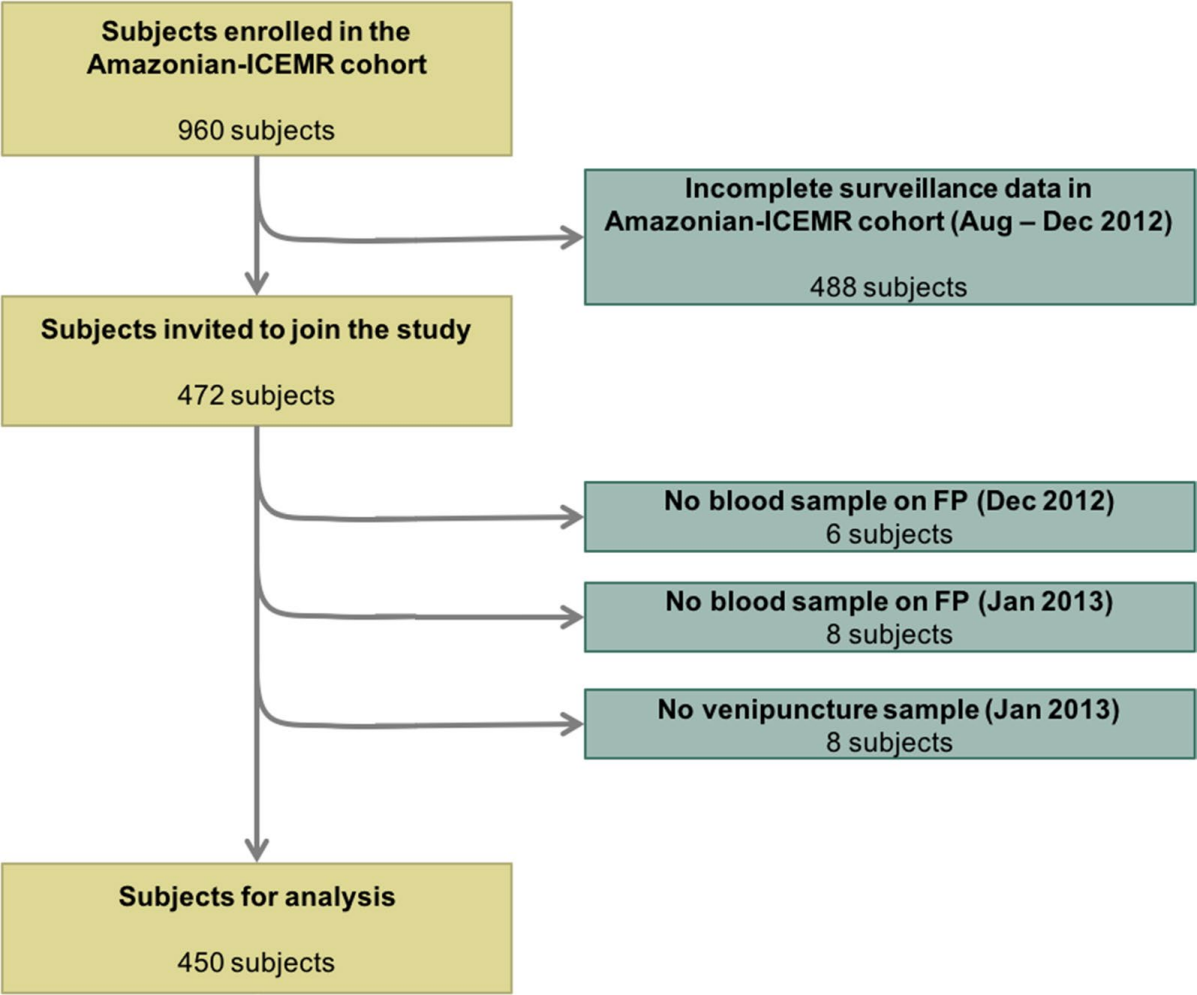
Flowchart of study participants. Based on selection criteria: complete surveillance (Aug–Dec 2012) in the Amazonian-ICEMR cohort, complete blood samples on filter paper (FP) on Dec 2012 and Jan 2013, and a venipuncture sample on Jan 2013

This study was carried out within the International Centers of Excellence for Malaria Research (ICEMR) Amazonia project based on a longitudinal, population-based cohort. For this study, a cross-sectional survey was conducted in January 2013, in which subjects with complete surveillance data from the Amazonian-ICEMR census (August 2012) were invited to participate. Of these, participants who consented to donate a blood sample by venipuncture were included in the study. The Amazonian-ICEMR cohort collected blood samples for light microscopy and on filter papers for PCR diagnosis. Participants with incomplete blood samples in December 2012 or January 2013 were excluded (Fig. 2).

### Data collection

A full census of the study population was conducted in August 2012. Individual and household data on socio-demographics (age, gender, education, occupation), self-reported previous history of clinical malaria, and structural characteristics of the household were collected. All households and inhabitants were encoded and geo-referenced using a Global Positioning System (GPS) handheld device (Garmin’s GPSMAP 60CSx, Garmin International Inc., USA).

During the cross-sectional survey conducted in January 2013, data regarding clinical examinations for fever and other malaria symptoms were recorded. Blood samples were taken by finger prick on slides for immediate microscopic examination (thick and thin blood smears). If a participant agreed, an additional 6 mL for adults or 3 mL for children of whole blood was collected by venipuncture in tubes with EDTA (BD Vacutainer, BD Franklin Lakes, USA) as preservative. Venipuncture blood samples were separated by centrifugation (3500 rpm) into plasma for serological analysis and packed red blood cells (PRBC) for molecular diagnosis. Both samples were, respectively, stored at 4 and − 70 °C until processed at the Institute of Tropical Medicine “Alexander von Humboldt”, Lima (ITM-AvH) for molecular (PCR) and serological (ELISA) analyses.

## Laboratory procedures

### Microscopy

Thick and thin smears were stained for 10 min with a 10% Giemsa solution (Giemsa’s azur eosin methylene blue, Merck KGaA, Germany) using a standard procedure [30]. Microscopic examination was immediately performed in the field by an expert microscopist after the sample collection. Parasite density for each species (parasites/μL blood) was determined by the number of parasites after counting a total of 200 white blood cells (WBC) and assuming an average of 6000 WBC/μL according to the national guidelines. Microscopy fields were read to count at least 500 WBCs before an individual was diagnosed as negative. Quality control was done blindly on all positive slides and 10% of randomly chosen negative slides by a senior technician at ITM-AvH.

### Molecular testing for malaria parasitaemia by PCR

PRBC samples were processed using the QIAamp DNA Mini Kit of QIAGEN (PRBC-PCR). For both sample types, subsequent amplification was performed by a real-time quantitative PCR (qPCR) method targeting the 18s rRNA gene region. Oligonucleotides 5-TAACGAACGAGATCTTAA-3 and 5-GTTCCTCTAAGAAGCTTT-3 were used as primers as reported by Mangold et al. [31] and the PCR conditions consisted of an initial denaturation at 95 °C for 2 min, followed by amplification for 45 cycles of 20 s at 95 °C, 20 s at 52 °C, and 30 s at 68 °C. Amplification was immediately followed by a melt programme consisting of 5 s at 65 °C and a stepwise temperature increase of 0.5 °C/s until 95 °C for species discrimination. Ambiguous melting results were confirmed by using a nested ssPCR method described elsewhere [32]. Parasite density was determined by using a standard curve from sevenfold serial dilutions to 1:10 of a culture sample at concentrations of 2 × 10^6^ parasites/µL down to 2 parasites/µL in blood from an uninfected donor.

### Serology

Recombinant MSP-10 proteins for *P. vivax* (PvMSP-10) and *P. falciparum* (PfMSP-10) (SalI and PF3D7 strains, respectively) were produced in HEK-293 mammal cells (Aragen Biosciences, California, USA). Plasma samples were analysed for total IgG reactivity to recombinant PvMSP-10 and Pf-MSP10 antigens by enzyme-linked immunosorbent assay (ELISA). 96-well ELISA plates (Clear Flat-Bottom Immuno, Thermo Scientific, USA) were coated with 0.5 μg/mL of the recombinant protein in 0.05 M carbonate buffer pH 9.6 overnight at 4 °C. Plates were washed 5 times with phosphate-buffered saline (PBS) containing 0.05% (0.3% for PfMSP-10) Tween 20 (PBST) and blocked for 1 h with 5% skimmed milk in PBST at room temperature. Plates were washed 5 times with PBST and both test and control plasma samples (1:500 diluted in blocking buffer) were added to each well in duplicate for 1 h at room temperature. A 1:10,000 dilution of peroxidase-conjugated goat anti-human IgG (Kirkegaard and Perry Laboratories, Inc., Gaithersburg, MD) was added to each well as a second antibody and incubated for 1 h. After three washings with PBST, bound antibodies were detected by adding the SureBlue tetramethyl benzidine substrate (Kirkegaard and Perry Laboratories, Inc., Gaithersburg, MD). The reaction was then stopped by the addition of 2 M H_2_SO_4_. Optical density (OD) at 450 nm was measured using an iMark^™^ microplate absorbance Reader (Bio-Rad Laboratories).

The corrected OD of each sample was calculated by subtracting the background OD from the corresponding non-coated wells. Serum samples from healthy non-exposed Peruvians were used as negative controls, and positive controls included samples from six different *P. vivax* and two *P. falciparum* infected individuals. A mixture model was used to determine OD cutoffs for the seropositivity of each *Plasmodium* spp. Briefly, mixture models allows to split the OD distribution into two Gaussian-distributed populations using a maximum likelihood approach (a narrow distribution of seronegatives and a broader distribution of seropositives). The cut-off to define seropositivity was the mean OD corresponding to the seronegative population plus 3 standard deviations [33–35].

### Statistical analysis

Statistical analyses were conducted in STATA 14 (StataCorp, 2015. Stata Statistical Software: Release 14. College Station, TX). The significance level was defined at 5 and 95% confidence intervals (CI) were estimated whenever appropriate. Fisher’s exact test was used for significance testing of categorical factors for each *Plasmodium* spp. and a negative binomial regression was used for significance testing of continuous skewed data (i.e. parasite density) [36]. All factors were obtained from the structured questionnaires or laboratory tests, and the report of a previous *Plasmodium* spp. infection detected by PCR for each participant was obtained from the Amazonian-ICEMR cohort data. Special attention was paid to time spent in the community; it was computed for permanent inhabitants (≥ 6 months living in the community) and a dummy variable was assigned according to recent (≤ 2 years) or long-term (> 2 years) settlement. This cut-off was set in order to evaluate the effect of malaria rebound since 2011 in the Peruvian Amazon Region [11, 29].

To handle the nested structure of sampled data—450 individuals nested within 235 households in 2 communities—a generalized linear mixed effects model (GLMM) was used. Two species-specific models were constructed with *Plasmodium* infection as the outcome. The dependent variable was defined as the PRBC-PCR (binary) result, and subjects with a different *Plasmodium* spp. Infection, detected by PRBC-PCR, were excluded in each model.

The univariate and multivariate analyses were fitted with a mixed-effects logistic regression, calculating odds ratios (OR). The suitability of the multilevel structure (two levels: individuals within households; or three levels: individuals within households within communities) was evaluated based on the variance components, intra-class correlation coefficient (ICC), and median odds ratio (MOR) of the null model [37]. The final multilevel structure was applied to both, univariate and multivariate regressions. Associated variables at *p* < 0.2 in the univariate mixed-effects logistic models for each *Plasmodium* species were included in its multivariate model construction. Final model variables were retained if *p* < 0.2 in a backward stepwise process.

## Results

### Socio-demographic and household characteristics

A total of 450 individuals (Fig. 2) from 252 households were enrolled in Cahuide (62%) and Lupuna (38%). Of these, 46% were male, and participants under 10, between 10 and 30, and over 30 years old represented 32, 39 and 30%, respectively. Just over half of adults (≥ 18 years) had primary school (53%) and the rest secondary school (47%) education. Most adults did not report a single specific occupation (51%), while 24% were farmers and 7% worked in the forest environment (loggers or charcoal workers). Finally, 16% of participants had recently moved and settled in the study areas (≤ 2 years) (Table 1).

**Table 1:** Baseline characteristics of the study population and their association with malaria infection by *P. vivax* and *P. falciparum*

Characteristics	*P. vivax* PRBC-PCR			*P. falciparum* PRBC-PCR			Total n = 450 (%)
	Positive n = 113 (%)	Negative n = 337 (%)	p value	Positive n = 22 (%)	Negative n = 428 (%)	p value	
Study area			0.146*			0.371	
Cahuide	77 (68.1)	203 (60.2)		16 (72.7)	264 (61.7)		280 (62.2)
Lupuna	36 (31.9)	134 (39.8)		6 (27.3)	164 (38.3)		170 (37.8)
Sex			0.191*			0.195*	
Male	58 (51.3)	148 (43.9)		7 (31.8)	199 (46.5)		206 (45.8)
Female	55 (48.7)	189 (56.1)		15 (68.2)	229 (53.5)		244 (54.2)
Age groups (years)			0.001**			0.022**	
< 10	24 (21.2)	119 (35.3)		2 (9.1)	141 (32.9)		143 (31.8)
10–29.9	41 (36.3)	133 (39.5)		9 (40.9)	165 (38.6)		174 (38.7)
≥ 30	48 (42.5)	85 (25.2)		11 (50.0)	122 (28.5)		133 (29.6)
Education			0.243			0.503	
None	10 (8.8)	51 (15.1)		1 (4.6)	60 (14.0)		61 (13.6)
Primary school	67 (59.3)	183 (54.3)		14 (63.6)	236 (55.1)		250 (55.6)
Secondary school or higher	36 (31.9)	103 (30.6)		7 (31.8)	132 (30.8)		139 (30.9)
Occupation (> 18 years old)			0.424			0.756	
Logger or charcoal worker	6 (9.0)	8 (5.8)		2 (13.3)	12 (6.3)		14 (6.9)
Farmer	19 (28.4)	30 (21.9)		3 (20.0)	46 (24.3)		49 (24.0)
Trader	4 (6.0)	8 (5.8)		0 (0.0)	12 (6.3)		12 (5.9)
Others	10 (14.9)	15 (11.0)		2 (13.3)	23 (12.2)		25 (12.2)
None	28 (41.8)	76 (55.5)		8 (53.3)	96 (50.8)		104 (51.0)
Time in community (years)			0.071*			0.209	
≤ 2	24 (21.8)	45 (14.2)		5 (26.3)	64 (15.7)		69 (16.2)
> 2	86 (78.2)	272 (85.8)		14 (73.7)	344 (84.3)		358 (83.8)
Mammal-livestock			0.443			1.000	
Yes	60 (53.1)	194 (57.6)		12 (54.5)	242 (56.5)		254 (56.4)
No	53 (46.9)	143 (42.4)		10 (45.5)	186 (43.5)		196 (43.6)
Avian-livestock			0.724			1.000	
Yes	77 (68.1)	236 (70.0)		15 (68.2)	298 (69.6)		313 (69.6)
No	36 (31.9)	101 (30.0)		7 (31.8)	130 (30.4)		137 (30.4)
Impregnated bed nets			0.048**			1.000	
Yes	75 (66.4)	257 (76.3)		16 (72.7)	316 (73.8)		332 (73.8)
No	38 (33.6)	80 (23.7)		6 (27.3)	112 (26.2)		118 (26.2)

*PRBC* packed red blood cells

Fisher’s exact test p value: * p < 0.2, ** p < 0.05

### Malaria parasite prevalence and exposure

Of the participants with complete surveillance data, a significant number of participants had previous *Plasmodium* spp. infection diagnosed by PCR on December 2012 (16% *P. vivax* and 2% *P. falciparum*). As of January 2013, 28 and 28% of subjects had evidence of recent exposure to *P. vivax* and *P. falciparum*, as determined by seropositivity to recombinant MSP-10.

The prevalence of *P. vivax* by PRBC-PCR (25%) was sevenfold higher than that determined by microscopy (3.6%). For its part, prevalence of *P. falciparum* by PRBC-PCR (5%) was 24-fold higher than that determined by microscopy (0.2%). The vast majority of subjects with any form of parasitaemia were asymptomatic; few participants (4%) had fever at the time of sampling (Table 2). No mixed-species infections were identified during the study.

**Table 2:** Prevalence of *P. vivax* and *P. falciparum* and its association with previous infection, exposure, symptoms and treatment

Characteristics	*P. vivax* PRBCs PCR			*P. falciparum* PRBCs PCR			Total n = 450 (%)
	Positive n = 113 (%)	Negative n = 337 (%)	p value	Positive n = 22 (%)	Negative n = 428 (%)	p value	
Positive PCR to *P. vivax* in previous month (Dec 2012)			0.017**			0.764	
Yes	26 (23.0)	45 (13.4)		4 (18.2)	67 (15.6)		71 (15.8)
No	87 (77.0)	292 (86.6)		18 (81.8)	361 (84.4)		379 (84.2)
Positive PCR to *P. falciparum* in previous month (Dec 2012)			1.000			0.081*	
Yes	2 (1.8)	8 (2.4)		2 (9.1)	8 (1.9)		10 (2.2)
No	111 (98.2)	329 (97.6)		20 (90.9)	420 (98.1)		440 (97.8)
Seropositivity to *P. vivax* (Jan 2013)			0.004**			0.221	
Yes	44 (38.9)	82 (24.3)		9 (40.9)	117 (27.3)		126 (28.0)
No	69 (61.1)	255 (75.7)		13 (59.1)	311 (72.7)		324 (72.0)
Seropositivity to *P. falciparum* (Jan 2013)			0.022**			0.340	
Yes	41 (36.3)	84 (24.9)		8 (36.4)	117 (27.3)		125 (27.8)
No	72 (63.7)	253 (75.1)		14 (63.6)	311 (72.7)		325 (72.2)
Positive microscopy to *P. vivax* (Jan 2013)			< 0.001**			1.000	
Yes	14 (12.4)	2 (0.6)		0 (0.00)	16 (3.7)		16 (3.6)
No	99 (87.6)	335 (99.4)		22 (100.0)	412 (96.3)		434 (96.4)
Positive microscopy to *P. falciparum* (Jan 2013)			1.000			0.049**	
Yes	0 (0.0)	1 (0.3)		1 (4.5)	0 (0.00)		1 (0.2)
No	113 (100.0)	336 (99.7)		21 (95.5)	428 (100.0)		449 (99.8)
Fever ^a^			0.247			0.185*	
Yes	6 (5.4)	10 (3.0)		2 (9.1)	14 (3.3)		16 (3.6)
No	105 (94.6)	322 (97.0)		20 (90.9)	407 (96.7)		427 (96.4)
Malaria treatment previous month			1.000			0.015**	
Yes	5 (4.4)	16 (4.7)		4 (18.2)	17 (4.0)		21 (4.7)
No	108 (95.6)	321 (95.3)		18 (81.8)	411 (96.0)		429 (95.3)

Based on PCR carried out on packed red blood cells

*PRBC* packed red blood cells

Fisher’s exact test p value: * p < 0.2, ** p < 0.05

^a^Factor with missing values

### Parasite density analysis

**Fig. 3:**
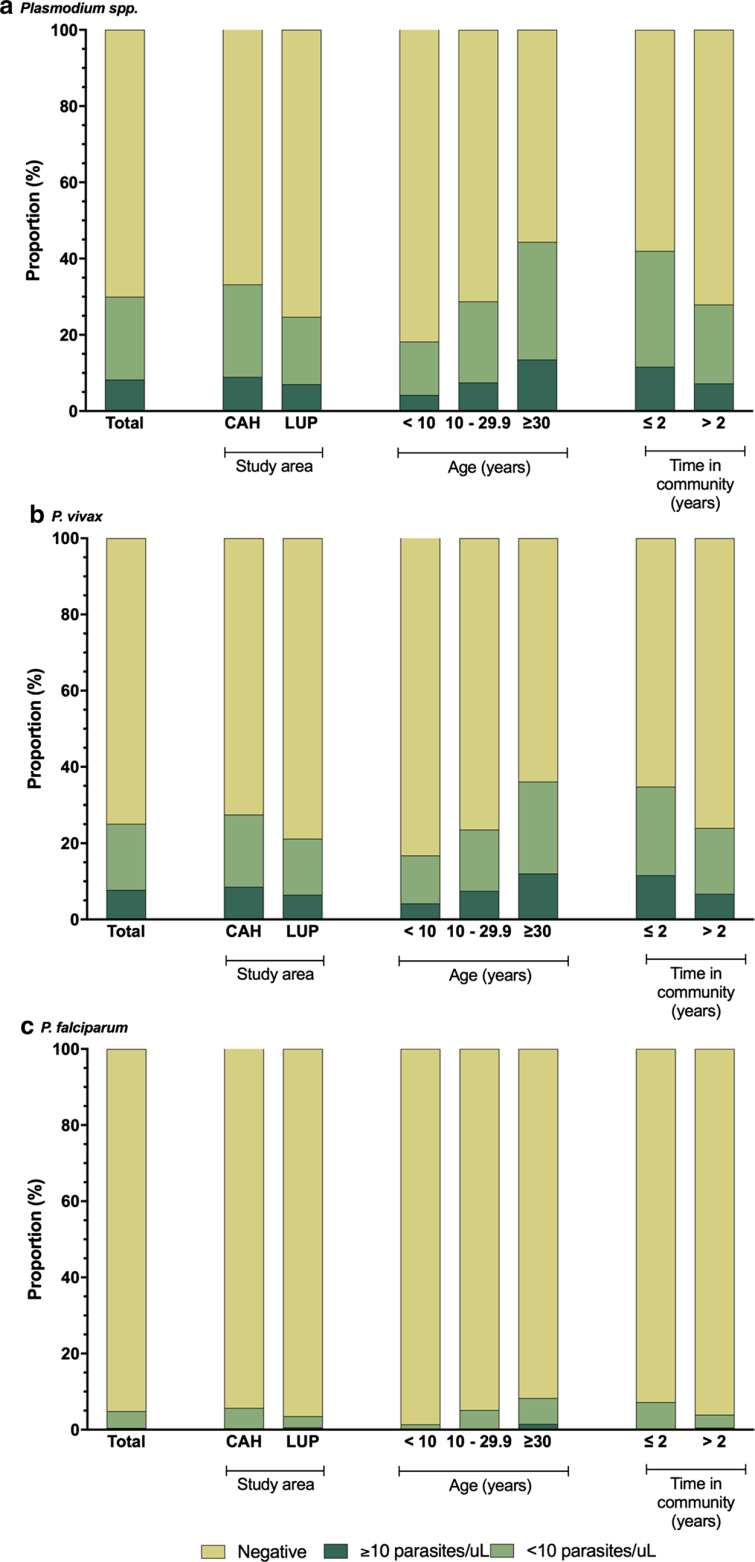
Stratified distribution of infections detected by packed red blood cells PCR (PRBC-PCR) according to epidemiological features (study area, age category and time in community) in Cahuide (CAH) and Lupuna (LUP), 2013. Colors indicates the proportion of positive infections with ≥ 10 parasites/µL (dark green), and < 10 parasites/µL (light green) for (**a**) *Plasmodium* spp., (**b**) *P. vivax* and ( **c**) *P. falciparum*

Parasite densities by PRBC-PCR were low (estimated geometric mean 57 parasites/μL, 95% CI 29–112). More than 40% of *Plasmodium* infections detected by PRBC PCR had fewer than 5 parasites/μL and 52% less than 10 parasites/μL. Important differences were observed between *P. vivax* and *P. falciparum* infections. The estimated geometric mean was higher in *P. vivax* (90 parasites/μL, 95% CI 42–190) compared to *P. falciparum* (8 parasites/μL, 95% CI 2–25). Parasite densities below 10 parasites/μL represented 50% of *P. vivax* and 68% of *P. falciparum* infections. The stratified distribution of infections with fewer than 10- and 5-parasites/μL according to some epidemiological features is presented in Fig. 3.

**Fig. 4:**
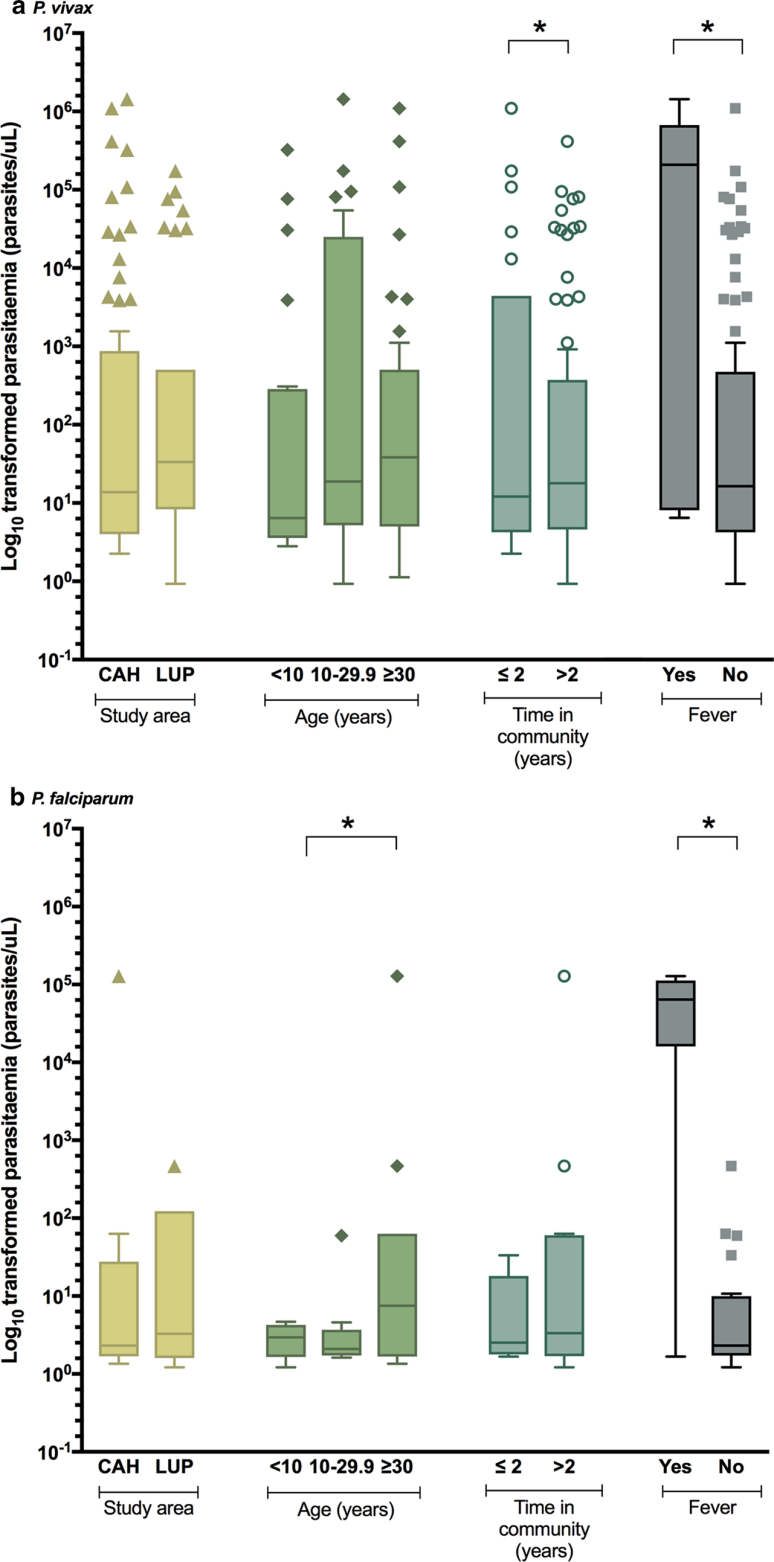
Parasite density of *Plasmodium* infections by packed red blood cells PCR (PRBC-PCR) according to epidemiological features (study area, age category, time in community and fever) in Cahuide (CAH) and Lupuna (LUP), 2013. Statistically significant differences in the multivariate negative binomial regression (asterisks) for (**a**) *P. vivax* and (**b**) *P. falciparum*. Estimates presented in Table 3

Recent immigrants to the area (≤ 2 years) had significantly higher logs of expected *P. vivax* parasite densities (coefficient 3.1; 95% CI 1.5:4.7; *p* < 0.01) compared to permanent settlers after adjusting for community, age, fever and seropositivity while for *P. falciparum* there were not statistically differences between immigrants and permanent settlers. For *P. falciparum*, the most important factor associated with a higher logs of expected *P. falciparum* parasite densities were fever (coefficient 6.4; 95% CI 4.3:8.4; p < 0.01) and seropositivity to PfMSP-10 (coefficient 3.1; 95% CI 1.7:4.6; p < 0.01), after adjusting for community, age and time in community (Fig. 4, Table 3).

**Table 3:** Estimates of the negative binomial regression for *P. vivax* and *P. falciparum* parasite densities

	*P.vivax*						*P. falciparum*					
	Univariate			Multivariate			Univariate			Multivariate		
	Coeff	95% CI	p value	Coeff	95% CI	p value	Coeff	95% CI	p value	Coeff	95% CI	p value
Study area (Ref = Cahuide)												
Lupuna	− 1.19	(− 2.37: − 0.01)	0.048*	0.99	(− 1.18:3.16)	0.371	− 4.6	(− 7.17: − 2.03)	< 0.001*	0.49	(− 0.96:1.95)	0.509
Age groups (Ref = < 10 years)												
10–29.9	0.93	(− 0.59:2.46)	0.230	0.46	(− 1.31:2.24)	0.606	1.08	(− 2.47:4.64)	0.550	1.10	(− 2.06:4.26)	0.495
≥ 30	0.57	(− 0.91:2.05)	0.449	− 0.28	(− 2.58:2.03)	0.814	8.28	(4.79:11.78)	< 0.001*	1.90	(− 1.36:5.15)	0.254
Time in community (Ref = > 2 years)												
≤ 2	1.67	(0.39:2.94)	0.010*	3.11	(1.50:4.71)	<0.001*	− 6.99	(− 9.66: − 4.32)	< 0.001*	− 0.68	(− 2.26:0.90)	0.401
Fever (Ref = No)												
Yes	2.94	(0.72:5.16)	0.009*	4.14	(1.12:7.17)	0.007*	7.55	(4.92:10.18)	< 0.001*	6.36	(4.28:8.43)	< 0.001*
Seropositivity to species-specific (Ref = No)												
Yes	8.40	(6.71:10.09)	<0.001*	− 0.46	(− 2.24:1.31)	0.608	8.61	(7.00:10.23)	< 0.001*	3.11	(1.66:4.56)	< 0.001*

Negative binomial models

*Coeff* Coefficient

Wald test p value, * p < 0.05

### Multilevel analysis for malaria infection

The hierarchical structure of data best fit with a multilevel structure with only two levels (individuals within households). Table 4 presents the results of the univariate and multivariate models for malaria infection with this multilevel structure for each *Plasmodium* species. Univariate analysis showed that participants over 30 years old (OR 3.7; 95% CI 1.9–7.4), seropositives to *P. vivax* (OR 2.3; 95% CI 1.3–3.8) and those with a *P. vivax* infection in the previous month (OR 2.1; 95% CI 1.1–3.8) were independently associated with *P. vivax* infection. On the other hand, only age (≥ 30 years, OR 9.7; 95% CI 1.8–51.5) was independently associated with *P. falciparum* infection.

**Table 4:** Fixed effects of univariate and multivariate multilevel logistic regression models of *P. vivax* and *P. falciparum* infections

	*P. vivax*						*P. falciparum*					
	Univariate			Multivariate			Univariate			Multivariate		
	OR	95% CI	p value	AOR	95% CI	p value	OR	95% CI	p value	AOR	95% CI	p value
Null model						< 0.001			< 0.001			< 0.001
Constant	0.33	(0.24–0.44)	< 0.001	0.13	(0.07–0.26)		0.07	(0.02:0.18)		0.01	(0.00:0.04)	
Study area (Ref = Cahuide)												
Lupuna	0.66	(0.40:1.11)	0.121				0.55	(0.21:1.44)	0.223			
Sex (Ref = Male)												
Female	0.75	(0.47:1.21)	0.239				1.75	(0.68:4.50)	0.242			
Age groups (Ref = < 10 years)												
10–29.9	1.81	(0.95:3.46)	0.070	1.73	(0.89:3.35)	0.103	4.47	(0.90:22.17)	0.067	10.72	(1.26:91.13)	0.030*
> = 30	3.74	(1.88: 7.43)	< 0.001*	3.31	(1.60:6.85)	0.001*	9.71	(1.83:51.52)	0.008*	16.32	(1.93:138.22)	0.010*
Education (Ref = None)												
Primary school	2.26	(1.00:5.11)	0.050*				4.20	(0.53:33.22)	0.174			
Secondary school or higher	2.22	(0.91:5.40)	0.077				3.65	(0.43:30.78)	0.234			
Occupation (Ref = Not a Logger, Charcoal worker or Farmer)												
Yes	1.70	(0.77: 3.75)	0.189				1.35	(0.43:4.24)	0.609			
Time in community (Ref = > 2 years)												
≤ 2	1.86	(0.97:3.55)	0.061	2.07	(1.03: 4.16)	0.042*	2.30	(0.79:6.74)	0.128	2.75	(0.88:8.59)	0.082
Mammal-livestock (Ref = No)												
Yes	0.83	(0.51:1.36)	0.459				0.88	(0.36:2.13)	0.776			
Avian-livestock (Ref = No)												
Yes	0.91	(0.53:1.54)	0.722				0.91	(0.36:2.33)	0.847			
Impregnated bed nets (Ref = No)												
Yes	0.60	(0.35:1.01)	0.054				0.82	(0.30:2.19)	0.689			
Fever^a^ (Ref = No)												
Yes	2.90	(0.80:10.56)	0.105				4.01	(0.69:23.20)	0.120	5.96	(0.98:36.18)	0.052
Seropositivity to species-specific (Ref = No)												
Yes	2.25	(1.34:3.78)	0.002*	1.82	(1.03: 3.20)	0.038*	1.80	(0.72:4.47)	0.206			
Positive PCR to species-specific in previous month (Ref = No)												
Yes	2.07	(1.14:3.80)	0.018*				5.46	(0.88:33.72)	0.067	10.40	(1.52:71.08)	0.017*
Malaria treatment previous month (Ref = No)												
Yes	1.09	(0.34:3.48)	0.888				5.61	(1.64:19.15)	0.006*			

Mixed-effects logistic models, only with random intercepts

*OR* odds ratio, *AOR* adjusted odds ratio

Wald test p value, * p < 0.05

^a^Factor with missing values

The multivariate analysis shows that participants with recent (≤ 2 years) arrival and settlement in the study area [Adjusted odds ratio (AOR) 2.1; 95% CI 1.03–4.2] had significantly higher odds of *P. vivax* infection, after adjusting for age (≥ 30 years, AOR 3.3; 95% CI 1.6–6.9) and seropositivity to *P. vivax* (AOR 1.8; 95% CI 1.03–3.2). Regarding *P. falciparum* infections, participants between 10 and 29 years (AOR 10.7; 95% CI 1.3–91.1) and ≥ 30 years (AOR 16.3; 95% CI 1.9–138.2) had significantly higher odds after adjusting for previous infection (AOR 10.4; 95% CI 1.5–71.1), time in community and fever symptoms (Table 4).

The random effects for each species-specific model are presented in Table 5. The ICC and the MOR of the adjusted models show an important clustering of *P. vivax* infections in households, an effect that was not observed in *P. falciparum*.

**Table 5:** Random effects of null and adjusted multilevel logistic regression models of *P. vivax* and *P. falciparum* infections fitted with 2 levels (individuals within households)

	Var		ICC		MOR
	Est.	95% CI	Est.	95% CI	
*P. vivax*					
Null model	0.7050	(0.2811:1.7686)	0.1313	(0.0235:0.4874)	1.9593
Adjusted model	0.7747	(0.2982:2.0131)	0.1543	(0.0263:0.5519)	2.0938
*P. falciparum*					
Null model	0.3465	(0.0001:1857.645)	0.0352	(0:1.0000)	1.3916
Adjusted model	0	(−)	0	(−)	1.0000

Mixed-effects logistic models; *var* variance estimated by mixed-effects model

*ICC* intra-class correlation coefficient, *MOR* median odds ratio

## Discussion

This study shows, consistent with previous studies, that malaria transmission in the Amazon Region is distributed in highly heterogeneous micro-geographic patterns, with the data suggesting that *P. vivax* transmitted more locally within villages and sources of *P. falciparum* more often being distantly acquired and transported on a regional basis. This conclusion is based on the observation that age 10 years and old, a surrogate for occupation-related riverine travel in our study population, is a strong, albeit non-exclusion risk factor for *P. vivax* vs. *P. falciparum* infection. While it is not possible to exclude entirely that some *P. vivax* transmission is acquired elsewhere and conversely that *P. falciparum* transmission occurs within home villages, the predominant pattern of transmission seems to follow the pattern that *P. falciparum* is mostly acquired away from and is reintroduced. Overall, the most important finding in this study was the demonstration that a large burden of sub-microscopic infections were detected in a traditionally low-transmission season. As a consequence, these results highlight the high proportion of *P. vivax* infections found in recent (non-immune) migrants, but that a higher proportion of *P. falciparum* infection was primarily found in subjects 10 and older, consistent with acquiring infection away from home, related to work. This study reported that relatively less-immune mobile people harbour higher *P. vivax* parasite densities while non-mobile village inhabitants harbour lower *P. vivax* parasite densities because they are more likely to develop clinical immunity due to local *P. vivax* transmission. In contrast, higher *P. falciparum* parasite densities were observed in inhabitants recently exposed to *P. falciparum* (PfMSP-10) and also with clinical symptoms, suggesting a lack of clinical immunity. These findings have direct relevance to malaria control and elimination strategies at the population level because they indicate that new public health strategies are required both to prevent local malaria transmission and the regional movement of parasites, particularly *P. falciparum.*

The sample collection method used in this study allowed for confident identification of associated factors to very low-level parasitaemia infections, which included, for *P. vivax,* age, time living in community and PvMSP10 seropositivity, and for *P. falciparum,* age and previous infections. The higher odds for *P. vivax* infections observed in migrant population (less than 2 years in the community) in comparison to permanent settlers suggest a certain level of naturally-acquired immunity to *P. vivax* due to local transmission exposure [38, 39]. These estimates were adjusted by a marker of recent exposure (PvMSP-10), hence the increased proportion of *P. vivax* infections observed in migrants participants was not an effect of recent exposure to parasite, but a lack of development of strain-specific acquired immunity. By contrast, the increased odds of *P. falciparum* infections according to age, instead of predominantly young people suggest that the local host population lacks immunity against the acquired *P. falciparum* parasites, for example as observed in imported malaria infections detected in the Solomon Islands [40, 41]. It is important to note that this increase in the *P. falciparum* infections was observed in populations over 10 years, the typically age where inhabitants in this study site start occupational related activities, and as consequence a high mobility outside community. In addition, the clustering of *P. vivax* infections at household level observed with the multilevel regression model, also support the hypothesis that *P. vivax* transmission occurs at local level. This household clustering pattern was observed in other studies with ongoing local malaria transmission [42–44]. Importantly, this effect was not observed for *P. falciparum* infections, suggesting a different transmission pattern for each *Plasmodium* species.

In areas with local malaria transmission, repeated exposure to infective mosquitoes’ bites carrying malaria parasites, leads to the fitness of the host’ immune system, and thus became both capable to control parasite density and clinical symptoms. Conversely, high parasite densities and clinical manifestations were observed as consequence to the exposition to a new parasite strain. Data reported here suggest that the differences in the parasite densities between *P. vivax* and *P. falciparum* presumably arose from the underlying transmission dynamic of each *Plasmodium* species. The higher *P. vivax* parasite densities in recent migrants compared to permanent settlers observed in this study is most consistent with a scenario of predominantly local, home-village-based *P. vivax* transmission. Inhabitants with recent exposure to *P. falciparum* (PfMSP-10) had higher parasite densities, suggesting a lack of immunity to *P. falciparum* strains possibly acquired outside communities due to occupational-related activities, as explained above. This important characteristic was not observed in *P. vivax*, because recent exposure to *P. vivax* (PvMSP-10) was not associated with high parasite densities. Relapses from hypnozoites could not be excluded as an explanation for the lack of association between parasite density and recent exposure. However, relapses in these areas presumably arose from infections in the recent past (4–10 weeks) [45–48]. Of note, albeit less marked than in this study, a high burden of sub-microscopic and very-low-densities infections has been reported in other settings in the Peruvian Amazon Region [6, 7, 11]. However, the results of this study show a parasite prevalence of 30% in a region characterized as low-transmission intensity. These findings challenge the traditional classification of the Peruvian Amazon as a hypoendemic malaria transmission setting [6] and indicate the existence of a sub-microscopic reservoir of infections between peaks of clinical malaria cases.

Taken together, the evidence presented here confirms previous data that malaria is distributed among scattered micro-geographic foci of infection in the Amazon Region [49]. This notion of spatially-constrained transmission is also supported by population genetics studies that report a strong population structure and high diversity and differentiation among communities [14, 50–52]. Studies on the *A. darlingi* biting behaviours also demonstrated that this vector, the most important in the Peruvian Amazon, has high behavioural plasticity [26, 53, 54], in particular animal biting preferences in diverse habitats [55–57], with biting taking place as far away as 400–500 m from breeding sites [58, 59]. In addition, remarkable differences in the human biting rate were reported at small- to moderate- spatial scales [10].

This study provides important insights into the contrasting transmission patterns of *P. vivax* and *P. falciparum* in the Peruvian Amazon context, particularly points of exposure, with relevance and potential generalizability to other low-transmission contexts. Therefore, future analytical approaches ought to focus on locations and populations at high-risk of *Plasmodium* species-specific infections where such infections co-exist. The data presented here suggest that optimal control of *P. vivax* transmission must address the interruption of transmission at the local level on subpatent parasitemics as well as hypnozoite carriers. Indoor residual spraying (IRS) and/or delivery of insecticide-treated bed nets (ITNs) are suitable adjunct control strategies due to highly-efficient sporogonic development of *P. vivax* within *Anophelines* [60]. Nonetheless, the hypnozoite reservoir remains a barrier to malaria control due to lack of suitable compounds for mass drug administration capable of eliminating hypnozoites. The most widely used drug to target hypnozoites is primaquine (PQ), but individuals with clinically significant glucose-6-phosphate dehydrogenase deficiency (G6PDd) have a high risk of haemolysis. In Peru, the Ministry of Health mandated treatment with PQ is not typically supervised [61], but the available evidence suggests low levels of clinically important G6PD mutations among Peruvians [62]. For its part, *P. falciparum* transmission needs a comprehensive framework to identify ‘source’ and ‘sink’ areas [63–65] to better allocate resources, and an importation vigilance in free-malaria areas to prevent reintroduction.

This study has some limitations. First, novel antigens (PvMSP-10 and PfMSP-10) with proven sensitivity for *P. falciparum* and *P. vivax* [66, 67] was used; however, local and regional variability in protective host immunes responses cannot be excluded as a contribution to the findings. Future studies with the use of more antigens as surrogate markers of clinical immunity [35, 68, 69] could address this limitation. Second, because this study was cross-sectional, associations with malaria status should be interpreted with caution as they do not imply causality. Still, the findings presented here are largely in agreement with previous work and local understanding of malaria transmission dynamics in this region. Third, the selection of participants was not based on a random sampling that would assure that missing data were completely at random (MCAR). However, since the exclusion of participants was not be due to having malaria or not, it is reasonable to assume that missing data was at random (MAR). While ideally MCAR is preferable, MAR re-assures us of a low likelihood of bias, at a level that would not mislead the data interpretation or conclusions. Furthermore, no statistically significant differences were observed between census demographics (sex, age, education, and occupation) of participants included and excluded in the present study. Finally, despite the shared vector, *A. darlingi*, in the Peruvian Amazon, no mixed infections were detected. This is likely due to several factors, including overall low transmission intensity, the micro-geographic localization of *P. falciparum*, and perhaps even intraspecific competition of both species within the human host. Finding no mixed infections is also possible because of fluctuating parasitaemias and the single point sampling of this study as well as technical limitations of the qPCR method at low-level parasitaemias. Thus, only one dominant *Plasmodium* species was observed in each test.

## Conclusions

This study highlights the varying transmission patterns of *P. vivax* and *P. falciparum* in the Peruvian Amazon Region. These findings suggest strong and stable local *P. vivax* transmission and imported *P. falciparum* transmission due to occupational-related activities. The detection of a high burden of very low parasitaemia carriers in this study opposes the traditional classification of the Peruvian Amazon Region as a hypo-endemic area and urges a reformulation of current malaria control policies.

## Abbreviations

MoH: Ministry of Health
PCD: passive case detection
PRBC: packed red blood cells
ICEMR: International Centers of Excellence for Malaria Research
ICC: intra-class correlation coefficient
MOR: median odds ratio
GLMM: generalized linear mixed effects model
CI: confidence interval
OR: odds ratio
Sen: sensitivity
Spec: specificity
Kc: Cohen’s Kappa coefficient
AOR: adjusted odds ratio
IRS: indoor residual spraying
ITN: insecticide-treated bed net
PQ: primaquine
G6PDd: glucose-6-phosphate dehydrogenase deficiency
